# The APAF1_C/WD40 repeat domain-encoding gene from the sea lettuce *Ulva mutabilis* sheds light on the evolution of NB-ARC domain-containing proteins in green plants

**DOI:** 10.1007/s00425-022-03851-0

**Published:** 2022-03-02

**Authors:** Michiel Kwantes, Thomas Wichard

**Affiliations:** 1 Institute for Inorganic and Analytical Chemistry, Friedrich Schiller University Jena, Lessingstr. 8, 07743 Jena, Germany; 2 Jena School for Microbial Communication, 07743 Jena, Germany

**Keywords:** Chlorophyta, Immunity, NBS-LRR, R-protein, Seaweed

## Abstract

**Main conclusion:**

**We advance** ***Ulva*’s genetic tractability and highlight its value as a model organism by characterizing its APAF1_C/WD40 domain-encoding gene, which belongs to a family that bears homology to R genes.**

**Abstract:**

The multicellular chlorophyte alga *Ulva mutabilis* (Ulvophyceae, Ulvales) is native to coastal ecosystems worldwide and attracts both high socio-economic and scientific interest. To further understand the genetic mechanisms that guide its biology, we present a protocol, based on adapter ligation-mediated PCR, for retrieving flanking sequences in *U. mutabilis* vector-insertion mutants. In the created insertional library, we identified a null mutant with an insertion in an apoptotic protease activating factor 1 helical domain (APAF1_C)/WD40 repeat domain-encoding gene. Protein domain architecture analysis combined with phylogenetic analysis revealed that this gene is a member of a subfamily that arose early in the evolution of green plants (Viridiplantae) through the acquisition of a gene that also encoded N-terminal nucleotide-binding adaptor shared by APAF-1, certain R-gene products and CED-4 (NB-ARC) and winged helix-like (WH-like) DNA-binding domains. Although phenotypic analysis revealed no mutant phenotype, gene expression levels in control plants correlated to the presence of bacterial symbionts, which *U. mutabilis* requires for proper morphogenesis. In addition, our analysis led to the discovery of a putative *Ulva* nucleotide-binding site and leucine-rich repeat (NBS-LRR) Resistance protein (R-protein), and we discuss how the emergence of these R proteins in green plants may be linked to the evolution of the APAF1_C/WD40 protein subfamily.

**Supplementary Information:**

The online version contains supplementary material available at 10.1007/s00425-022-03851-0.

## Introduction

Chlorophyte and streptophyte algae (green algae) are diverse oxygenic photosynthetic eukaryotes that obtained their chloroplasts through an ancient endosymbiotic event with a cyanobacterium [McFadden 2001; and reviewed in Graham et al. (2009); Gawryluk et al. 2019]. Our understanding of their evolutionary relationships has greatly benefited from recent advances in phylogenomics, which retraced, for example, the trajectory by which land plants evolved from streptophyte algae (Turmel et al. 2006; Wodniok et al. 2011; Timme et al. 2012) and discerned the existence of three phyla in the clade of green plants (Viridiplantae) (Li et al. 2020).

Green algae are important to the global carbon cycle and local ecosystems as primary producers. Although some of them may also occupy terrestrial, subaerial niches, streptophyte algae are regarded as strict freshwater lineages, whereas chlorophyte algae have adapted to oceanic and coastal marine environments, as well as freshwater (Becker and Marin 2009). In addition to the various ecosystems in which chlorophyte algae have settled, the different lineages are also characterized by highly variable morphologies, ranging from motile single cells to sessile multicellular organisms with body plans consisting of multiple tissues (Leliaert et al. 2007). Because of the well-resolved green algal phylogeny, it has been established that the transition from unicellularity to multicellularity took place on multiple independent occasions in both streptophyte and chlorophyte algae (Niklas and Newman 2013; Umen 2014; Del Cortona et al. 2020). Similarly, it is likely that streptophyte algae conquered land multiple times during the course of evolution [reviewed in Delwiche and Cooper (2016) and Fürst-Jansen et al. (2020)], while in the chlorophyte lineage, freshwater to marine transitions (or vice versa) frequently occurred (Dittami et al. 2017).

To better understand the intriguing biology of chlorophyte algae several model organisms have been established; for example, the single-celled *Chlamydomonas reinhardtii* and the multicellular colonial alga *Volvox carteri*, which have been valuable for understanding photoreceptor biology and morphological complexity, respectively (Nagel et al. 2002; Matt and Umen 2016). Given the enormous diversity in green algal biology, it is remarkable, however, that the insight into the molecular mechanisms that guided chlorophyte algae evolution is still relatively limited in comparison to our understanding of streptophyte algae and land plants.

To empower further research on chlorophyte algae, several genome sequencing initiatives have been undertaken. Recently, also the genome of the sea lettuce *Ulva mutabilis*, a multicellular chlorophyte alga belonging to the class Ulvophyceae that is abundant in intertidal zones worldwide (Wichard et al. 2015), was published (De Clerck et al. 2018). The body of *U. mutabilis* consists of a flat distromatic blade connected via a stem-like structure to a holdfast that can attach to the stratum. Within the genus, tubular forms also exist, and *Ulva* species generally display a high degree of phenotypic plasticity, which has made the unification of its taxonomy challenging (Steinhagen et al. 2019). *Ulva* species are also characterized by their ability to proliferate quickly under high nutrient conditions and are therefore infamous for producing blooms, or green tides, in eutrophicated waters, which can be harmful to local economies and ecosystems (Valiela et al. 1997; Smetacek and Zingone 2013). Conversely, under normal conditions, *Ulva* species are important ecosystem organizers that contribute to the habitat of numerous coastal dwellers and thus sustain biodiversity (Mann 1973). In addition to their role in the intertidal zone, *Ulva* species, as well as other marine algae, are increasingly appreciated as a sustainable source for animal feed, plant fertilizer and various biomaterials, and in processes as integrated multi-trophic aquaculture and the bioremediation of wastewater (Bikker et al. 2016).

*U. mutabilis* was developed as a laboratory organism in the mid-1900s. Pioneering studies unraveled its life cycle, in which isomorphic haploid and diploid phases alternate, but also showed that unfertilized gametes could form parthenosporophytes via autodiploidization (Føyn 1958; Hoxmark 1975). Additionally, the fast-growing mutant “*slender*” was isolated, which can be maintained in the haploid phase, thereby simplifying genetic analysis (Føyn 1959). *U. mutabilis* has furthermore received considerable attention because of its dependence on specific bacterial symbionts, or the compounds they produce, to undergo proper morphogenesis and development (Spoerner et al. 2012), a trait it shares with the related alga *Monostroma oxyspermum* (Monostromataceae, Ulvales) (Matsuo et al. 2005). Central to both species is their reliance on the compound thallusin, which *U. mutabilis* acquires from the epiphytic bacterium *Maribacter* sp. MS6 (Alsufyani et al. 2020) and requires for rhizoid development and regular cell wall formation. In addition to *Maribacter* sp. (or thallusin), *U. mutabilis* morphogenesis also depends on the activity of *Roseovarius* sp. MS2, which releases compounds that promote cell division and blade formation. Only under the synergistic effect of the compounds produced by both *Roseovarius* sp. and *Maribacter* sp., *U. mutabilis* will develop properly (Spoerner et al. 2012; Grueneberg et al. 2016).

Recently, the genetic tractability of *U. mutabilis* (Oertel et al. 2015) has been boosted by the development of a molecular toolkit that improved and broadened the strategies for ectopic gene expression and the targeting of proteins to specific cellular compartments (Blomme et al. 2021). These transformation tools also allow for the generation of mutant libraries by integrating foreign DNA into the *Ulva* genome. One of the most critical steps after constructing a random insertion mutant library is the identification of the insertion sites, which often is laborious, but a variety of techniques has been developed for the efficient retrieval of the regions that flank insertion sites (Tam and Lefebvre 1993; Liu et al. 1995; O'Malley et al. 2007; Sun et al. 2019). Together, these methods have proven very successful for elucidating gene function in land plants and green algae (Alonso et al. 2003; Dent et al. 2005). Hence, functional gene studies based on mutant analysis in *U. mutabilis* could aid in harnessing its agronomic potential (Charrier et al. 2015) and help answer, for example, whether the genetic networks that evolved in complex chlorophyte algae resemble those from streptophyte and other archaeplastidal lineages.

To accommodate genetic analysis in *U. mutabilis*, we present in this study an adapter ligation-mediated PCR method that allowed us to efficiently map several flanking regions in a collection of insertional mutants. Moreover, in this library, we identified a null mutant in an APAF1_C/WD40 repeat domain-encoding gene. Further phylogenetic and protein domain architecture analysis was used to track the fate of this gene family, thereby also shedding light on the evolution of NB-ARC domain-containing proteins in the green lineage.

## Materials and methods

### Plant material and cultivation

For all experiments, the *U. mutabilis* laboratory strain “*slender*” was used, unless indicated otherwise. Plants were cultivated in *Ulva* culture medium (UCM) (Stratmann et al. 1996; Wichard and Oertel 2010) in a growth chamber with a long-day (17 h light/7 h dark) light regime (~ 30 µmol photons m^−2^ s^−1^) at 18 °C. For phenotyping of transgenic plants, axenic cultures were prepared according to Califano and Wichard (2018) and subsequently inoculated with *Roseovarius* sp. strain MS2 and/or *Maribacter* sp. strain MS6 (Spoerner et al. 2012).

### Creation of a library of *Ulva mutabilis* transgenic lines

Polyethylene glycol-mediated transformation was performed according to the protocol described in Oertel et al. (2015) and subsequently genotyped according to Boesger et al. (2018). In brief, before transformation, gametes were obtained from induced *Ulva* gametophytes, purified by phototactic migration through Pasteur pipettes (Califano and Wichard 2018) and transformed using SspI-linearized vector pPIBT7 (GenBank EU176859.1), which carries a codon-optimized phleomycin-resistance gene expression cassette as a selection marker. Transformed gametes were selected on 50 µg ml^−1^ phleomycin in *Ulva* culture medium incubated for 4 days. After about 4 weeks of cultivation in UCM, selected surviving gametophytes were individually grown in tissue culture flasks. For genotyping, a tissue sample (up to 100 mg per plant) was used for genomic DNA extraction using the GenElute Plant Genomic DNA Miniprep Kit (Merck). Genotyping PCR was performed using Prime-STAR GXL DNA Polymerase (Takara) in the presence of 5% DMSO using a primer pair specific for the resistance cassette and as a control for the gene *UM008_0183*. Oligomers are listed in Suppl. Table S1.

### Identification of insertion sites by adapter ligation-mediated PCR

To create a template suited for PCR, genomic DNA (gDNA) containing the pPIBT7 insert was digested with a restriction enzyme and adapters were ligated to the overhanging ends of the gDNA. A short strand and a long strand make up the adapter. When the two strands of the adapter are hybridized, the short strand has a 5′ overhang, which is complementary to the restriction half-site ‘sticky end’ of the gDNA and will anneal to it; subsequently, the fragments are fused by ligation (O'Malley et al. 2007). Approximately 100 ng of gDNA was double-digested by the restriction enzymes BamHI/BclI (New England Biolabs), producing a 5′ GATC overhang. The digested gDNA was subsequently purified with the Nucleospin Gel and PCR Clean-up Kit (Macherey–Nagel) and ligated to a compatible adapter, present in a calculated 20-fold excess of DNA ends, that was made by annealing oligomers p46 and p48. The adapters were largely based on those used by Thole et al. (2009) but sequence-optimized to reduce aspecific amplification during PCR. They were created by mixing 6.25 µl of each adapter oligo (100 µM) in a total volume of 50 µl 1 × restriction enzyme buffer solution to provide positively charged ions to accommodate annealing. Subsequently, the solution was heated for 2 min at 95 °C and cooled gradually over 45 min. To amplify flanking sequences, a nested PCR approach was performed using the non-proofreading enzyme TAKARA taq (Takara) according to the manufacturer's instructions, but in the presence of 5% DMSO. The first PCR consisted of 20 cycles using the oligos p39/30 on ~ 35 ng adapter-gDNA template. The second, nested PCR consisted of 32 cycles using oligo p47/34 on 1 µl of the reaction products from the first PCR. Alternatively, the primer combinations p39/52 and p47/43 can be used for the first and nested PCR. Products from the second PCR were loaded on a 2% agarose gel and stained using HD-Green Plus (Intas). Amplicons that appeared as distinct bands were cut out and gel purified to serve directly as templates for Sanger sequencing. The resulting sequence reads were screened for the presence of a vector-flanking region border, and the identified flanking regions were analyzed by blasting them against the annotated wild-type *U. mutabilis* genome sequence (Sterck et al. 2012; De Clerck et al. 2018) (see also https://bioinformatics.psb.ugent.be/orcae/). Oligomers are listed in Suppl. Table S1.

### cDNA cloning and qPCR expression analysis

The cDNAs of wild-type *UM033_0004* and *UM005_0337* were cloned using the SMARTer RACE 5′3′ Kit (Takara). First, total RNA was extracted using the Spectrum plant total RNA kit, including the optional on-column DNase I digestion (Merck). Then, (nested) 5′RACE was used to determine the 5′UTR region, and subsequently, a forward primer in the 5′UTR was designed for 3′RACE of the full-length transcript, which was cloned in pJET1.2 (Thermo Fisher) and sequenced. Normalized relative expression of *UM033_0004* and *UM005_0337* was determined using real-time quantitative PCR (qPCR) using the reference genes *UM008_0183* (Ubiquitin) and *UM010_0003* (PP2A 65 kDa regulatory subunit A). For qPCR, 1 µg of total RNA was reverse transcribed using the SuperScript IV VILO Master Mix Reagents (Thermo Fisher). Subsequent qPCR was done using PowerUp SYBR green chemistry (Thermo Fisher) on a CFX96 Real-Time PCR Detection System (Bio-Rad). The Cq values and melting curves were analyzed with CFX Maestro qPCR Analysis Software that implements the Pfaffl method (Pfaffl 2001; Vandesompele et al. 2002). Amplification efficiencies were determined from the dilution series. Additionally, qPCR amplicons were analyzed on a 2.5% agarose gel, and direct sequencing of the amplicons verified specific cDNA amplification. RACE and qPCR primers are listed in Suppl. Table S1.

### Sequence identification and protein domain analysis

Initial protein domain analysis of UM033_0004 was performed using the online portals SMART (Letunic et al. 2020) (http://smart.embl-heidelberg.de) and Interproscan (Blum et al. 2020) (https://www.ebi.ac.uk/interpro). Chlorophyte homologs of UM033_0004 were identified by performing a blastp query (Altschul et al. 1997; Camacho et al. 2009; Cock et al. 2015) against a BLAST database that was built from all published chlorophyte algae proteomes (filtered models) available on Phycocosm (Grigoriev et al. 2020). All hits (0.001 *e*-value cutoff) were selected and then filtered for the presence of the APAF1 helical domain (APAF1_C, PF17908/IPR041452) or its corresponding gene3D signature (G3DSA:1.25.40.370). To identify homologs from more distantly related taxa, hidden Markov model (HMM) searches were performed (Finn et al. 2011). A custom APAF1_C HMM profile was generated (hmmbuild) from an alignment (hmmalign) of all the chlorophyte UM033_0004 homologs identified, against the Pfam APAF1_C HMM profile. Subsequently, separate HMM searches were performed against the following Joint Genome Institute datasets: Streptophyta (including the embryophyte genomes from *Marchantia polymorpha, Physcomitrella patens, Sphagnum fallax* and *Arabidopsis thaliana*), the prasinodermophyte *Prasinoderma coloniale*, the glaucophyte *Cyanophora paradoxa* and Rhodophyta. Complete species lists and data sources are listed in Suppl. Table S2. Also, an additional HMM search against all the streptophyte algae included in the One Thousand Plant Transcriptomes Initiative project was performed (Carpenter et al. 2019). For the identification of non-archaeplastidal eukaryote and eubacterial sequences, we used the HMM search tool from the EBI (Potter et al. 2018) with our custom APAF1_C profile. The presence of APAF1_C, WD40 (IPR001680), WH-like (IPR036388), NB-ARC (IPR002182), and LRR (IPR032675) domains was investigated via Interproscan ( Zdobnov and Apweiler 2001; Quevillon et al. 2005; Hunter et al. 2008; Cock et al. 2015) based on the Pfam release 34.0, (Mistry et al. 2020). Tools from the Galaxy webserver were used for all analyses (Afgan et al. 2018) unless indicated otherwise. All identified sequences are presented in Supplementary Data File S1.

### Phylogenetic analysis

We included UM033_0004 and all the chlorophyte and streptophyte homologs identified in the phylogenetic analysis, except those that encoded only the APAF1_C domain. Additionally, from the HMM searches against Rhodophyta, non-archaeplastidal eukaryotes and eubacteria, the top 2–4 sequences (from different species) with the lowest *e*-value were included, as well as Apoptotic human protease activating factor 1 (APAF1). To root the phylogenetic tree, we also aligned the WD40 repeat domain of the fungal protein HET-E, which contains an N-terminal NACHT-domain ATPase. An initial protein alignment was created against the custom APAF1_C HMM profile, whereas the C-terminal part, containing the WD40 repeats, was aligned using MUSCLE (Edgar 2004). Sequences N-terminal to the APAF1_C domain were not aligned. Multiple sequence alignment and maximum likelihood analysis was performed using the software package MEGA7 (Kumar et al. 2016), using the substitution model LG + G + F. All aligned positions were considered with a cutoff of 85% (678 positions). The protein alignment with annotated domains can be found in Supplementary Data File S2.

## Results

### Adapter ligation PCR retrieves four unique pPIBT7 insertion sites

**Fig. 1:**
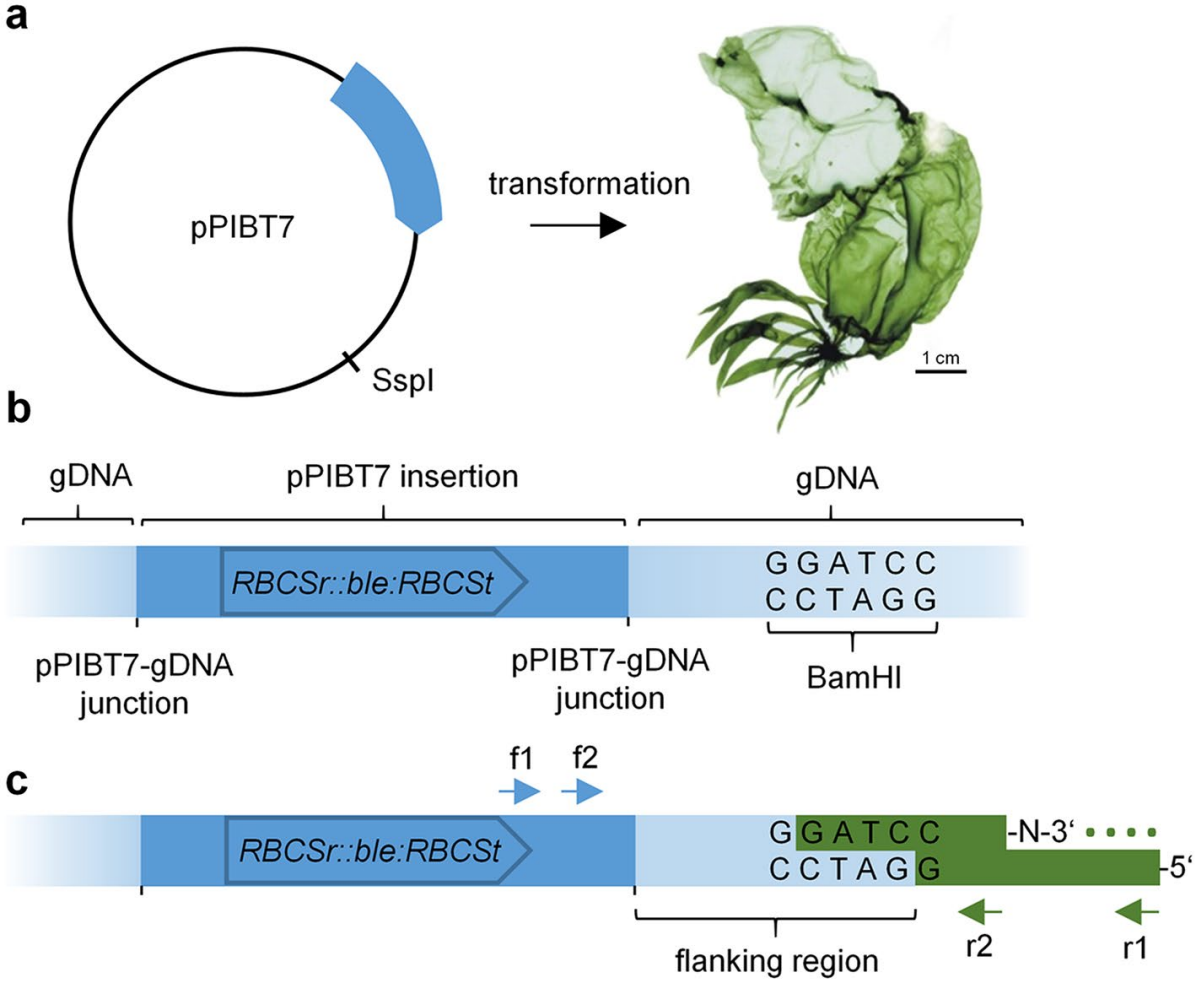
Schematic overview of adapter-ligation PCR approach. **a** For insertional mutagenesis, pPIBT7 carrying the phleomycin-resistance marker *RBCSr::ble:RBCSt* was linearized using SspI and transformed into *Ulva*, thereby creating genomic insertion sites as schematically shown in **b**. **c** Digestion of genomic DNA (gDNA) using BamHI (and BclI, not shown) leaves 5'GATC overhangs to which a compatible asymmetric adapter is ligated. The adapter 3′ amino-terminal group prevents DNA polymerase extension of the adapter sequence; consequently, the annealing site (dotted line) for the reverse primer (r1, arrow) only becomes available after the successful extension of the pPIBT7 insert specific primer f1 (arrow). A nested PCR using primers f2 and r2 further ensures the specific amplification of vector-gDNA segments. Arrows indicate primers. Sequences are not drawn to scale. Photography of *U. mutabilis* was reprinted from Wichard and Oertel (2010) with permission from JohnWiley and Sons, Copyright© (2010) Wiley

To establish a method to retrieve vector-insertion flanking sequences from *U. mutabilis*, we first created a collection of insertional mutants via polyethene glycol-mediated vector transformation. After selection and genotyping, an adapter-ligation PCR approach (Fig. 1) (O'Malley et al. 2007; Thole et al. 2009) was adopted to recover the sequences flanking the vector-insertion sites. Notably, we needed to modify the adapter sequences established by Thole et al. (2009) because the corresponding primers generated high background amplification when tested on genomic DNA extracted from untransformed control plants. Additionally, we found that adapter-ligation PCR required a polymerase without 3′–5′ proofreading activity because this exonuclease activity seemed incompatible with the presence of a C7 amino modification at the 3′ end of the short adapter oligo. Ultimately, single flanking sequences were identified from each of four plants, whereas two plants both yielded two distinct sequences (Table 1). Notably, the insertion site could not be pinpointed in four cases because the BLAST search using the retrieved flanking sequence as a query returned multiple hits in the *Ulva* genome, indicating that these insertions were probably in repetitive sequences. Of the other sites, two were situated in intergenic regions and two in predicted introns. In summary, we identified eight insertion sites from six transgenic lines (Table 1).

**Table 1:** Overview of transgenic lines and the retrieved genomic insertion sites

Transgenic line/insert	Locus identifier (*Ulva* wt v1)	Region	Distance to locus	Protein annotation
7.11	*UM033_0004*	Intron	–	WD40-repeat-containing
7.12	*UM008_0094*	Intergenic	516 bp downstream of stop codon	TLDc-domain-containing
7.14	No unique blast hit	–	–	–
7.20	No unique blast hit	–	–	–
7.24 insert 1	*UM005_0337*	Intron	–	Membrane-bound protein
7.24 insert 2	No unique blast hit	–	–	–
7.25 insert 1	*UM010_118*	Intergenic	1507 bp upstream of start codon	Cobalamin-independent methionine synthase
7.25 insert 2	No unique blast hit	–	–	–

### Molecular characterization of the *UM033_0004* and *UM005_0337* insertional mutants

**Fig. 2:**
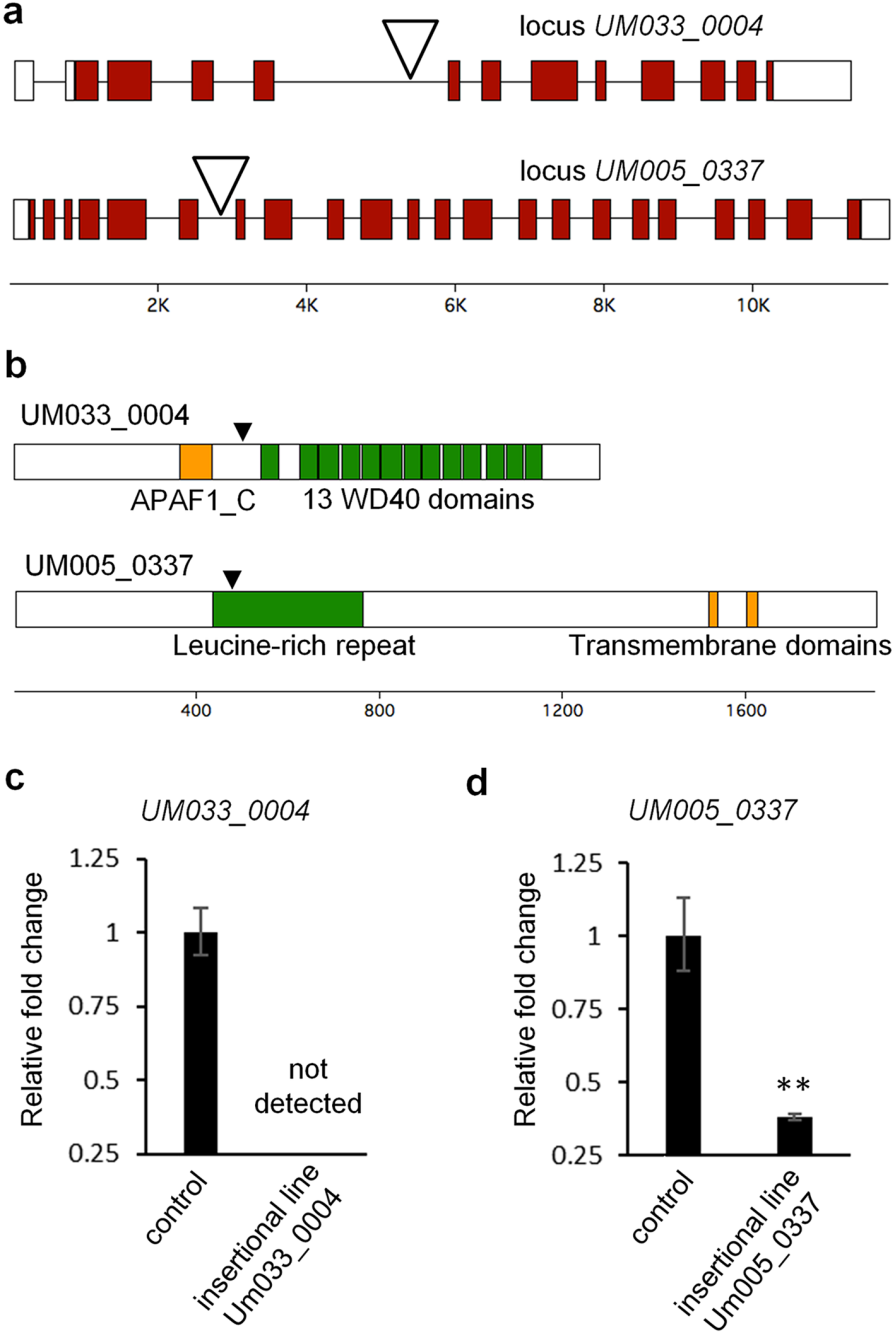
Molecular characterization of the *Ulva UM033_0004* and *Um005_0337* insertional mutants. **a** Exon–intron structure and position of the pPIBT7 insertion. Shaded boxes represent exons, white boxes represent UTRs and lines introns. Open triangles indicate the insertion sites. **b** Domain architecture of the proteins corresponding to loci *UM033_0004* and *UM005_0337*. Closed triangles indicate the positions where the pPIBT7 insertions interrupt the corresponding transcripts. **c** Transcript abundance of the identified genes in the insertional mutant lines and untransformed *Ulva* “*slender*” control plants. Error bars show the standard error of the mean (*n* = 3). N.D., not detectable. **, Student’s *t* test *P* < 0.01

Next, we characterized the insertion sites in the predicted introns in more detail by amplifying and sequencing the vector-gDNA junctions using pairs of gene-specific primers, which verified their location in a WD40 repeat-encoding gene (*UM033_0004*, see Suppl. Fig S1) and in a membrane-bound protein-encoding gene (*UM005_0337*) that were also identified in the initial BLAST search (see Fig. 2a, b). Subsequently, the exon–intron structures of the loci were determined by identifying the 5′UTR using 5′RACE and subsequently cloning the cDNAs via 3′RACE. Sequence analysis showed that both genes followed the annotated exon–intron structure that corresponds to the coding sequence. Interestingly, in the case of *UM033_0004* we additionally identified an intron in the 5′UTR and a 3′UTR of more than 1000 base pairs (Fig. 2a).

Real-time quantitative PCR (qPCR) revealed the effect of the intronic insertions on the accumulation of the gene transcripts. Using primer pairs that anneal downstream or on opposite sides of the insertions, no transcript of the WD40 repeat-encoding gene could be detected (Fig. 2c), and transcript levels of the membrane-bound protein-encoding gene were reduced approximately threefold (Fig. 2d). Therefore, although both insertions are located in introns, the insertion in the *UM005_0337* locus seems to only partly downregulate the production of a mature mRNA, whereas the transcription or pre-mRNA processing of the WD40 repeat-encoding gene appears to have been disrupted, thereby creating a null mutant.

### UM033_0004 has homology to NB-ARC domain-containing proteins

**Fig. 3:**
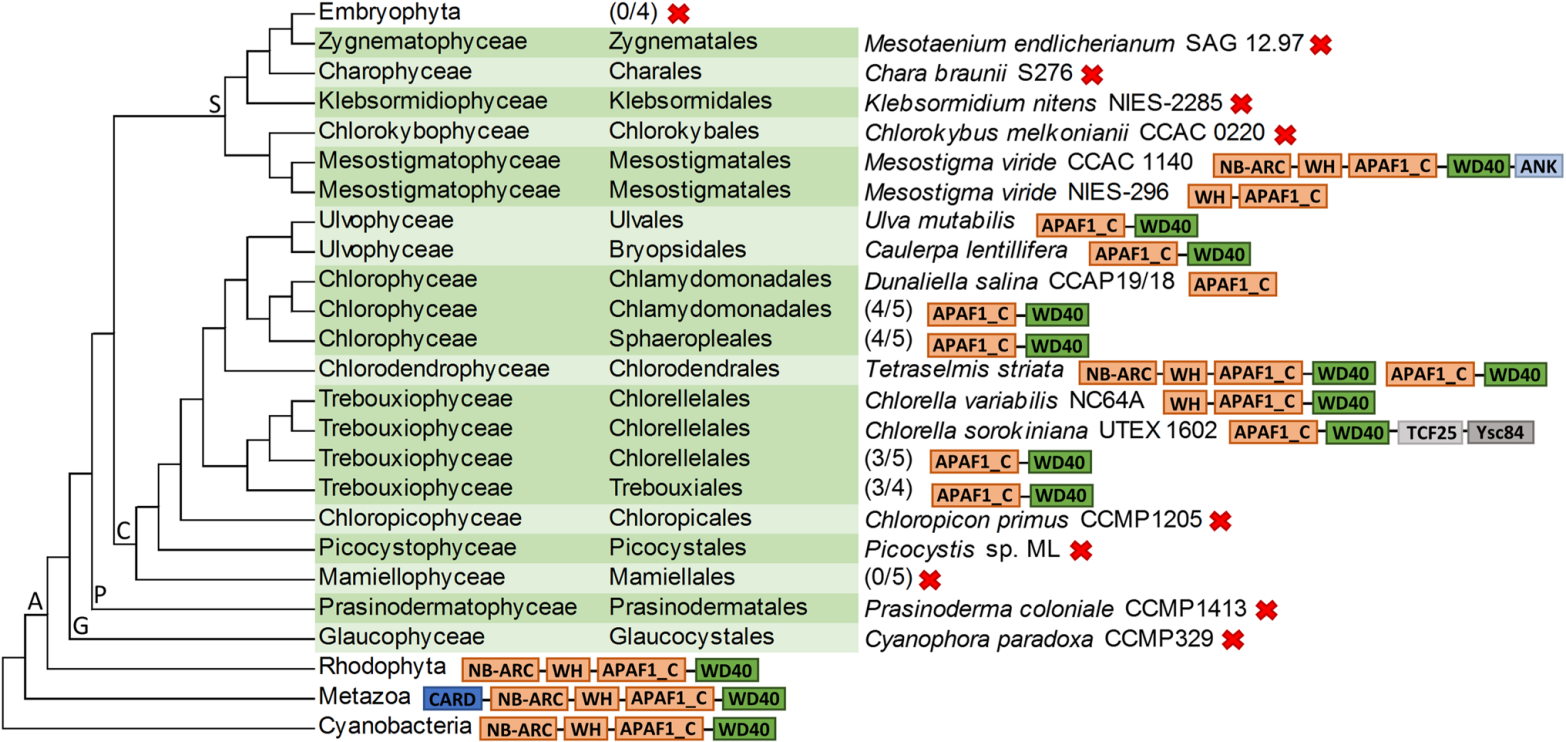
Cladogram showing the domain architectures of the retrieved APAF1_C/WD40 domain-encoding genes and the phylogenetic relationship of the lineages in which they were identified. The relationship among Archaeplastida lineages (A) follows Li et al. (2020). The position of Picocystophyceae basal to Chloropicophyceae is according to Turmel et al. (2019). For Streptophyta (S) (except Embryophytes), Chlorophyta (C), Prasinodermaphyta (P) and Glaucophyta (G), the taxonomic class and order of the studied species are according to AlgaeBase (Guiry and Guiry 2021). The nomenclature of *C. melkonianii* follows the recent revision of the Chlorokyboceae by Irisarri et al. (2021). Numbers in brackets are fractions of the total number of species (some species include multiple accessions, see Supplementary Table S2) in which the depicted APAF1_C/WD40 module was positively identified, whereas red crosses indicate that no homologs were identified in the corresponding lineage. Protein domain abbreviations: ANK (Ankyrin repeat-containing domain), APAF1_C (apoptotic protease activating factor 1 helical domain), CARD (caspase activation and recruitment domain), NB-ARC (nucleotide-binding adaptor shared by APAF1, certain R-gene products and CED-4), TCF25 (transcription factor 25), WD40 (WD40 domain-containing repeat), WH (winged helix-like DNA-binding domain superfamily). Protein domain lengths are not to scale

As a complete knock-out mutant was established for *UM033_0004*, we further investigated its potential function using protein domain analysis. Genome annotation and the SMART protein domain architecture tool indicated it contained 13–15 WD40 repeats. InterProScan additionally identified the presence of the APAF1_C domain located N-terminally to the WD40 repeat domain (see Fig. 2b). Interestingly, Pfam HMM profile analysis revealed that *UM033_0004* is the only *Ulva* gene that encodes this domain, while there are a total of 156 genes that matched the WD40 profile. The specific domain architecture of UM033_0004 prompted us to investigate the distribution of any homologs among the group of Archaeplastida by systematically performing similarity searches in a total of 41 taxa, including 25 chlorophyte algae, 5 streptophyte algae, 4 embryophytes, 1 prasidermophyte, 1 glaucophyte and 6 rhodophyte species, which were accessed via the Phycocosm and Phytozome genome repositories. Within the phylum of chlorophyte algae, proteins containing this module were detected in all investigated classes of core chlorophytes (Ulvophyceae, Chlorophyceae, Chlorodendrophyceae and Trebouxiophyceae) (Fig. 3). In *Dunaliella salina* (Chlorophyceae), we identified two proteins that only contained the APAF1_C helical domain. In contrast, two out of four *Tetraselmis striata* (Chlorodendrophyceae) homologs possessed both an additional N-terminal NB-ARC and a domain resembling the WH-like DNA-binding domain superfamily. In one out of three investigated *Chlorella* species, the N-terminal WH-like domain was detected, too, and in one case, accessory C-terminal transcription factor 25 (TCF25) Ribosome quality control complex subunit 1 in yeast) and the actin-binding domain Ysc84 domains were also present. Among the other major groups of Viridiplantae, searches using the HMM profile did not detect the APAF1_C domain in the investigated Embryophyte, Prasinodermophyte or Glaucophyte proteomes However, it was present in the Mesostigmatophyceae (Streptophyta) and Rhodophyta sequences. The retrieved *Mesostigma viride* CCAC 1140 and the rhodophyte proteins displayed N-terminal NB-ARC and WH-like domains, resembling the domain architecture observed in two of the *T. striata* proteins. However, the *M. viride* CCAC 1140 protein had an additional C-terminal ankyrin domain repeat, whereas the *M. viride* NIES-296 sequence only contained the WH-like and APAF1_C domains. A similar HMM search against the streptophyte algal proteomes included in the One Thousand Plant Transcriptomes Initiative did not deliver any further homologs. The HMM hits with the lowest *e*-value from non-archaeplastidal eukaryotes belonged to basal groups of metazoans, whereas the most significant bacterial hits were cyanobacterial. Interestingly, in these proteins, the APAF1_C/WD40 module was linked to NB-ARC and WH-like domains as in *T. striata* and *M. viride* CCAC 1140, and in the metazoan homologs, it was additionally linked to an N-terminal caspase activation and recruitment (CARD) domain, which is typical for APAF1-like proteins (Hofmann and Bucher 1997).

### Phylogenetic analysis reveals that *UM033_0004* is a member of a green algal APAF1_C/WD40 domain repeat-encoding gene subfamily

**Fig. 4:**
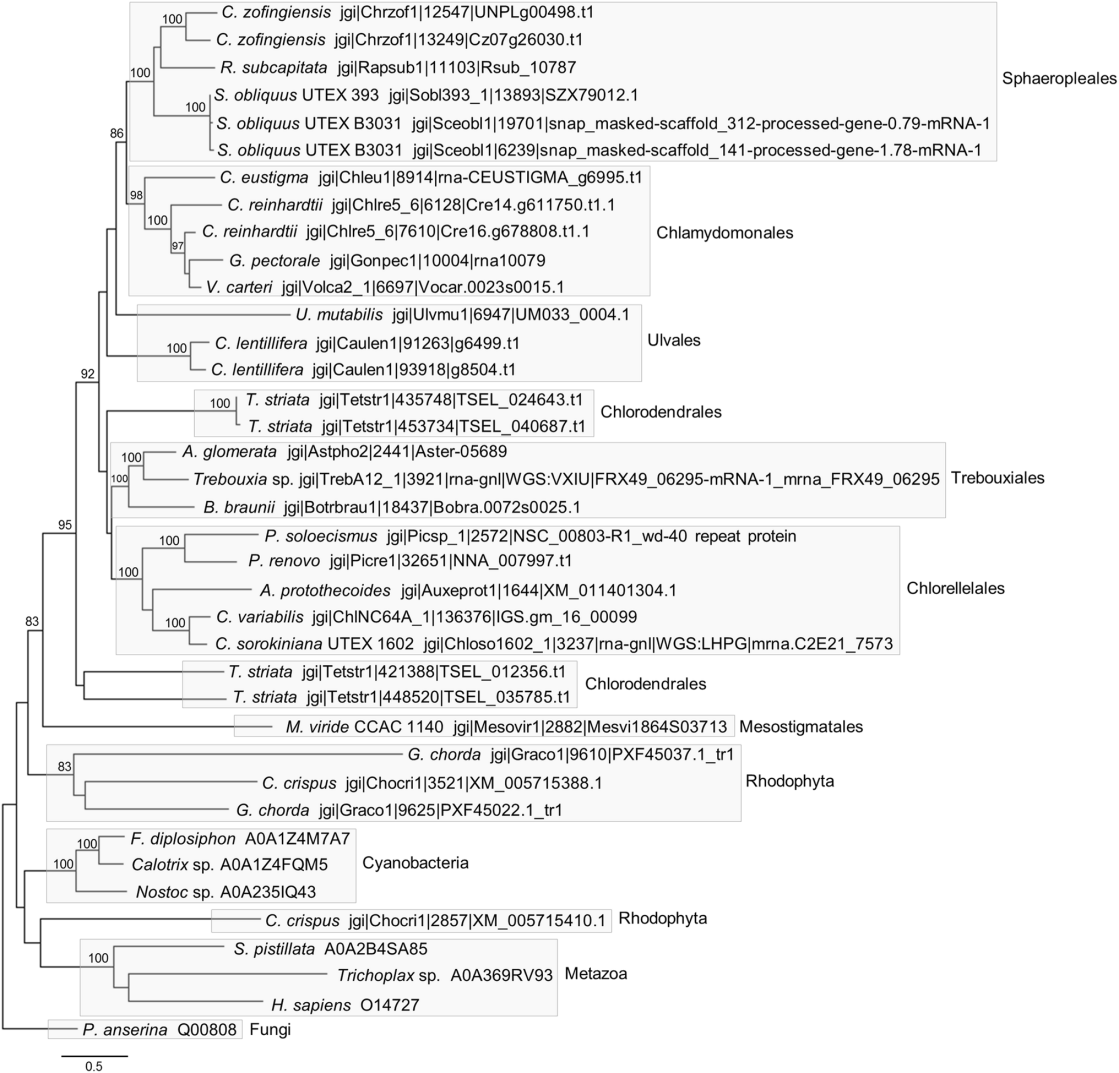
Maximum-likelihood phylogenetic tree using the LG + G + F substitution model based on the alignment of UM033_0004 and 37 identified homologs. Only the aligned APAF1_C/WD40 domain-containing regions were included (678 positions) with a gap cutoff of 85% (see Suppl. Data File 2). Bootstrap values (500 resamplings) > 80% are shown. The scale bar indicates the number of amino acid substitutions per site. Sequences are represented by the name of the organism from which they originated and the sequence identifier (see also Suppl. Data File S1). The NB-ARC domain-containing *T. striata* proteins have the identifiers TSEL_012356.t1 and TSEL_035785.t1

To study the evolutionary relationship between the identified proteins, we created a multiple sequence alignment of their APAF1_C and WD40 domains. We added to the alignment the human homolog APAF1, and the WD40 repeat domain from the fungal protein HET-E, which was later used to root the phylogenetic tree. Maximum-likelihood analysis gave rise to highly supported clades that corresponded to groups of metazoa, cyanobacteria and chlorophyte/streptophyte algae, whereas the red algal sequences did not form a single clade (Fig. 4). Additionally, all Chlorophycea sequences clustered together, with proteins from the orders Chlamydomonadales and Sphaeropleales forming subclades within this group, and the sequences from the orders Chlorellales and Trebouxiales formed distinct subgroups. However, the interrelationships between these groups and the representatives from the orders Chlorodendrales, Bryopsidales and Ulvales were not entirely resolved. Although other routes cannot be excluded, the clear separation of the green algal proteins from the other groups suggests a common evolutionary origin of all the chlorophyte and streptophyte APAF1_C/WD40 domain-containing proteins.

Furthermore, the occurrence and distribution of the N-terminal NB-ARC and WH-like domains among representatives of both algal lineages aligns with a parsimonious scenario in which these domains were inherited from an NB-ARC/WH-like/APAF1_C/WD40 domain-containing protein present in the ancestor of these lineages. Interestingly, the identification of the cyanobacterial sequences (Fig. 3), which mirrored such a domain architecture and the architecture of the rhodophyte sequences, hinted at a vertical inheritance of a NB-ARC/WH-like/APAF1_C/WD domain-encoding gene via the ancestral cyanobacterial endosymbiont. However, a close relationship between the proteins from cyanobacterial, rhodophyte, and chlorophyte/streptophyte sequences was neither supported nor rejected by the phylogenetic analysis (Fig. 4). Notably, in the cyanobacterium *Gloeomargarita lithophora*, the closest extant relative of plastids (Ponce-Toledo et al. 2017), we could not detect any sequences that matched either our custom chlorophyte or the Pfam APAF1_C HMM profile. Therefore, as is being considered for the metazoan APAF1 homologs (Urbach and Ausubel 2017), the putative ancestral chlorophyte/streptophyte NB-ARC/WH-like/APAF1_C/WD40 repeat domain-encoding gene might also have been acquired through lateral gene transfer.

### Survey of NB-ARC domain-containing proteins in *Ulva*

The evolutionary loss of the N-terminal NB-ARC domain of the potentially ancestral NB-ARC/WH-like/APAF1_C/WD40 protein in the lineage to *Ulva* and other chlorophyte algae, could likely have drastic effects on the function of this protein. Therefore, we considered that the *Ulva* APAF1_C/WD40 protein might form a functional complex with an NB-ARC domain encoded on a separate locus. However, a survey of the *Ulva* proteome did not identify any solitary NB-ARC domains, which we envisaged as candidates for such a role. Interestingly, the survey did identify an NB-ARC domain associated with a tetratricopeptide repeat (UM014_0006.1) and an NB-ARC domain-containing protein (UM003_0200.1) with C-terminal LRR-domains (Suppl. Fig. S2). Therefore, the latter protein is likely a representative of the NBS-LRR protein family that is involved in immunity (van der Biezen and Jones 1998).

### Assessment of the role of *Ulva*’s APAF1_C/WD40 protein

**Fig. 5:**
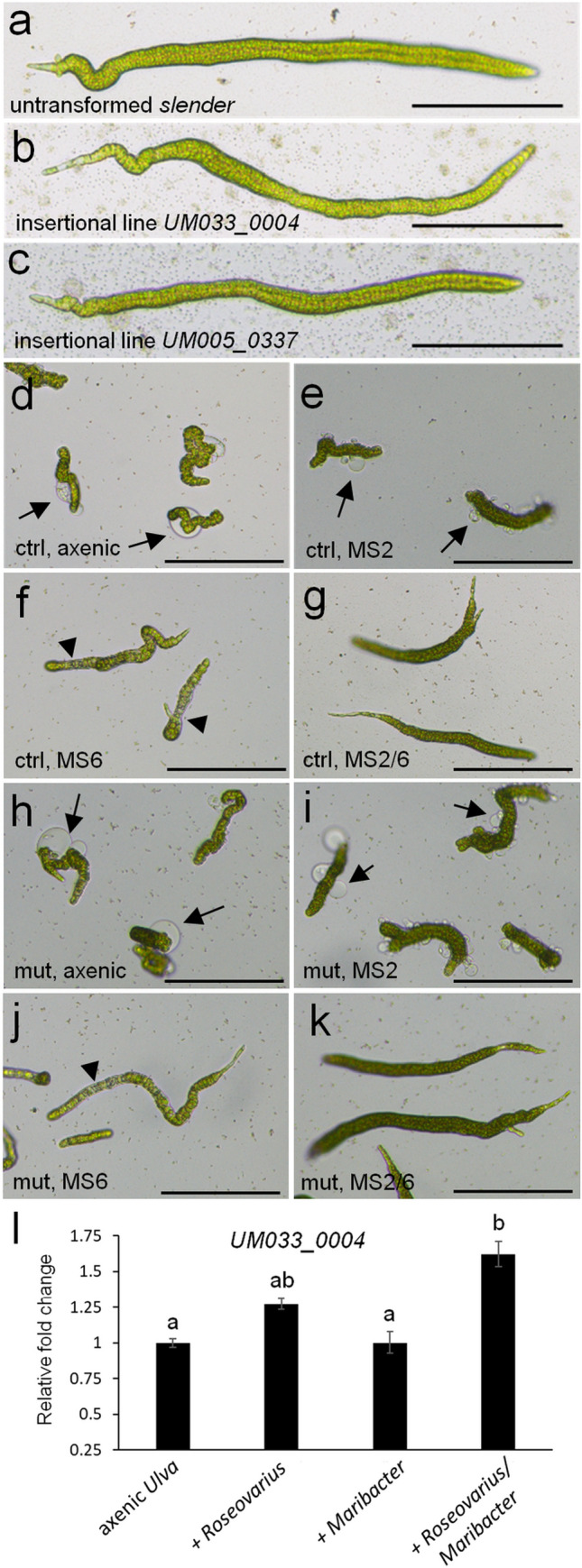
Phenotypic analysis of insertional mutants and transcript abundance of *UM033_0004* under different bacterial regimes. Phenotypes of representative individuals of untransformed “*slender*” (**a**), insertional line *UM033_0004* (**b**) and *UM005_0337* (**c**) 12 days after gamete release and grown in tissue culture flask in the presence of their bacterial symbionts. Phenotypes of untransformed “*slender*” under axenic conditions (**d**) or in the presence of *Roseovarius* sp. MS2 only (**e**), *Maribacter* sp. MS6 only (**f**), or in the presence of both *Roseovarius* sp. MS2 and *Maribacter* sp. MS6 (**g**) after 18 days of growth in a 96-well plate. Phenotypes of insertional mutant *UM033_0004* under axenic conditions (**h**) or in the presence of *Roseovarius* sp. only (**i**), *Maribacter* sp. only (**j**), or in the presence of both *Roseovarius* sp. and *Maribacter* sp. (**k**) after 18 days of growth in a 96-well plate. **l** Relative transcript abundance of *UM033_0004* in *Ulva* “*slender*” under different symbiont regimes. Statistical significance was inferred using a one-way ANOVA. Treatments not sharing the same letter are significantly different (Tukey HSD test, *P* < 0.01). Error bars show the standard error of the mean (*n* = 3). Arrows highlight bubble-like protrusions that occur in the absence of *Maribacter* sp., triangles indicate degenerate cells. Size bar is 200 µm

To examine the effect of the insertions in *UM033_0004* (APAF1_C/WD40 domain-encoding) and *UM005_0337* (membrane-bound protein-encoding) on the phenotype of the mutant plants, we propagated the primary transformants and grew new generations of gametophytes from unfertilized gametes. Microscopic inspection of germlings 12 days after gamete release did not show apparent phenotypic alterations compared with untransformed control plants (Fig. 5a–c). In the case of *UM005_0337*, this could suggest that the degree of downregulation achieved by the insertion (Fig. 2d) is insufficient to evoke a phenotypic change, although it may also be redundant with other genes. For the *UM033_0004* null mutant (Fig. 2c), redundancy is unlikely because protein domain analysis indicate it is a single-copy gene. Therefore, *UM033_0004* might be required only under specific external conditions. Because *Ulva* development largely depends on the presence of two bacterial symbionts that have complementing and synergetic effects (Spoerner et al. 2012; Grueneberg et al. 2016), we also investigated the phenotypes of *UM033_0004* mutant and control plants under different microbial regimes. These bioassays corroborated the specific deficiencies when one or both of the symbionts (*Roseovarius* sp. strain MS2 and *Maribacter* sp. strain MS6) are missing, as previously observed (Spoerner et al. 2012), but it did not expose a difference between the control (Fig. 5d–g) and mutant plants (Fig. 5h–k). However, an examination of transcript abundance in control plants that were grown under identical conditions showed that *UM033_0004* transcript levels were reduced when one or both of its symbionts were absent (Fig. 5l). Transcript reduction was equally large in the absence of *Roseovarius* sp. MS2 alone or in the absence of both symbionts, whereas in the absence of *Maribacter* sp. MS6 only, transcript levels were reduced to intermediate levels.

## Discussion

The recent increase in the availability of chlorophyte and streptophyte algae genome sequences has broadened the scope of plant comparative and functional genomics (Blaby-Haas and Merchant 2019). As a result, there is an incessant demand for molecular tools tailored to each prospective model organism to explore the role of individual genes experimentally. In the recently sequenced multicellular seaweed *U. mutabilis*, a transformation method that provides the means to create mutants through vector insertion is already available (Oertel et al. 2015), but had not yet resulted in the production of verified gain- or loss-of-function mutants. In the present study, we build on this technology by showing that it is possible to pinpoint the location of genomic vector-insertion events upon vector transformation by adopting an adapter-ligation PCR approach. Moreover, among the insertional mutants that were characterized, a null mutant was identified. This demonstrates the feasibility of future forward genetic screens in *Ulva* to successfully identify the casual insertional mutation, which promises to be more cost- and time-efficient than positional cloning or sequencing-based strategies.

Identifying the *UM033_0004* null mutant in our insertional library encouraged us to investigate further the potential role of this gene and its evolutionary history. Protein domain architecture combined with phylogenetic analysis indicated that the *Ulva UM033_0004* gene is a member of an APAF1_C/WD40 repeat-encoding gene family that was probably founded early in the evolution of the chlorophyte/streptophyte lineage by the acquisition of a gene that also encoded N-terminal NB-ARC and WH-like domains. This scenario implies that members of this family have been independently lost in several individual species of chlorophyte algae and the class of Mamiellophycean algae, as well as after the clade of Mesostigmata/Spirotaenia/Chlorokyboceae (from which we analyzed the genome sequence of one *C. melkonianii* and two *M. viride* accessions) split from the other streptophytes. The latter event also explains the absence of this gene family in the investigated land plants. The resulting scattered distribution indicates that the cooption of the genes was highly selective rather than that they played a role common to green algae.

A comprehensive study of signal transduction NTPases with numerous domains proteins by Urbach and Ausubel (2017) showed a close relationship between NB-ARC domain-containing plant R proteins, which have C-terminal LRR repeats and are involved in immunity (van der Biezen and Jones 1998), and the metazoan pro-apoptosis factor APAF1-like proteins that possess C-terminal WD40 domain repeats (Cecconi 1999). The functioning of both classes of proteins depends on NB-ARC domains that can oligomerize and build geometric protein complexes, while the repeat domains perform a sensory role (Reubold et al. 2011; Wang et al. 2019). The loss of the likely ancestral N-terminal NB-ARC- and WH-like domains in many chlorophyte lineages, including *Ulva*, suggests that this gene family acquired a restricted or specialized role in these lineages during evolution that solely depends on the APAF1_C/WD40 domain repeat. Because the WD40 repeats fold into beta-propellers that promote protein–protein interactions (Mishra et al. 2014), its ability to serve as a scaffold or interact otherwise probably dictates the function of the APAF1_C/WD40 protein in *Ulva* and other chlorophyte algae. Interestingly, structural studies of the metazoan APAF1 protein, which is an essential pro-apoptosis factor, have shown that the APAF1_C subdomain [described as helical domain 2 (HD2)] (Riedl et al. 2005; Dorstyn et al. 2018) serves as a rigid arm that positions the WD40 beta-propeller relative to the NB-ARC and WH-like domains. On the other hand, the WH-like domain can make various inter-and intramolecular contacts with the NB-ARC domain (Dorstyn et al. 2018). Therefore, the conserved association of the APAF1_C domain, and in some cases the WH-like domain, to the WD40 repeat suggests that they may assist in configuring the spatial orientation of any interacting molecules. Further identification of the interaction partners will be the next step to understanding the molecular function of the *Ulva* APAF1_C/WD40 protein.

Because of the homology of the chlorophyte APAF1_C/WD40 protein with APAF1-like proteins, and because chlorophyte algae also possess regulated cell death mechanisms (Moharikar et al. 2007; Bidle 2016), it is conceivable that the NB-ARC/WD40 gene acquired by an ancestral chlorophyte was integrated into a similar pathway as the metazoan APAF1-like proteins. However, phenotypic analysis of the *UM033_0004* knock-out under normal conditions or in the absence of one or both of its symbionts did not reveal a mutant phenotype. Interestingly, transcript abundance levels showed a pattern that reflects the synergistic nature of the growth-promoting role of *Ulva*'s symbionts. Further functional studies of *UM033_0004* may thus aid in understanding the genetic networks underlying bacteria-dependent morphogenesis in *Ulva*.

In addition to these results, our research also revealed the existence of a plant-type NBS-LRR R-protein-encoding gene in the *Ulva* genome. Only recently the existence of such proteins, which in land plants are important in host–pathogen interactions, has been determined in streptophyte algae (Urbach and Ausubel 2017; Gao et al. 2018) as well as the chlorophyte algae *Chromochloris zofingiensis* (class Chlorophyceae) and two *Botryococcus braunii* accessions (class Trebouxiophyceae) (Shao et al. 2019). The discovery of an additional R-protein in *Ulva* (class Ulvophyceae) therefore considerably strengthens Shao et al.’s conclusion (2019) that a protein with an NBS-LRR architecture was already present in the common ancestor of the entire green lineage. However, the exact origin and molecular mechanisms that led to the emergence of this ancestral plant R-protein are still under debate. It has been suggested that NBS-LRR proteins evolved through an NB-ARC/WD40 domain-containing intermediate (Urbach and Ausubel 2017), but the closest relative of the R-protein family was not determined, nor was a protein family encoding an NB-ARC/WD40 module identified in the green lineage. Our analysis has now shown that a gene family which encoded these domains was likely present early in the evolution of green plants. Therefore, if NB-ARC/WH-like/APAF1_C/WD40 proteins predate R proteins, R proteins might not have gained their NB-ARC domain from a foreign source (Andolfo et al. 2019). Instead, R proteins could have emerged in an ancestral green alga through a genetic recombination event between endogenously available NB-ARC- and LRR-encoding genes. Finally, it will be fascinating to discover which effector molecules interact with algal R proteins, from which (microbial) organism they originate, and if these molecules are the result of harmful or symbiotic interactions. In addition to effector-triggered immunity, pattern-triggered immunity involving receptor-like kinases (Gong and Han 2021) is also an important mediator of plant immunity. Further investigations of both modes of action, which evolved in parallel (de Vries et al. 2018; Gong and Han 2021) will likely deepen our understanding of the nature of early plant–microbe interactions.

## Conclusions

In summary, our research offers a technical advance in the genetic tractability of *Ulva mutabilis*. We provide a methodology for obtaining flanking sequences from *U. mutabilis* vector-insertion mutants using adapter ligation-mediated PCR. A null mutant was identified in an APAF1 C/WD40 repeat domain-encoding gene. Although no mutant phenotype was observed, gene expression levels in control plants were linked with the presence of bacterial symbionts, which *U. mutabilis* requires for its morphogenesis. Moreover, in addition to defining a green algal family of APAF1_C/WD40-containing proteins, our research revealed a likely plant-type NBS-LRR R-protein-encoding gene that provided insight into the evolution of plant R proteins, thereby underlining the value of *Ulva* as a reference model organism.

### *Author contribution statement*

TW and MK conceived the study and interpreted the results. MK performed the genotyping and all following experiments, analyzed the data and wrote the first draft of the manuscript. TW and MK contributed to the final version of the manuscript and act both as corresponding authors.

## Supplementary Information

Below is the link to the electronic supplementary material.

Supplementary file1 (XLSX 45 KB)

Supplementary file2 (XLS 2656 KB)

Supplementary file3 (DOCX 228 KB)

## Data Availability

The data for this study have been deposited in the European Nucleotide Archive (ENA) at EMBL-EBI under accession number PRJEB46783 (https://www.ebi.ac.uk/ena/browser/view/PRJEB46783).

## Abbreviations

APAF1: Apoptotic protease activating factor 1
APAF1_C: Apoptotic protease activating factor 1 helical domain
HMM: Hidden Markov model
LRR: Leucine rich repeat
NB-ARC: Nucleotide-binding adaptor shared by APAF-1, certain R-gene products and CED-4
NBS-LRR: Nucleotide-binding site and leucine-rich repeat
WH-like: Winged helix-like
