# Supplementary material for: The APAF1_C/WD40 repeat domain-encoding gene from the sea lettuce Ulva mutabilis sheds light on the evolution of NB-ARC domain-containing proteins in green plants
Source: Planta. 2022 Mar 2;255(4):76. doi: 10.1007/s00425-022-03851-0 (PMC8891106; doi:10.1007/s00425-022-03851-0)
Supplement: Supplementary file 3 — Supplementary file3 (DOCX 228 KB) [file 425_2022_3851_MOESM3_ESM.docx]

**Supplementary material for**

**The APAF1_C/WD40 repeat domain-encoding gene from the sea lettuce *Ulva mutabilis* sheds light on the evolution of NB-ARC domain-containing proteins in green plants**

Michiel Kwantes ^1,^* and Thomas Wichard ^1,2,^*

^1^ Institute for Inorganic and Analytical Chemistry, Friedrich Schiller University Jena, Lessingstr. 8, Jena, 07743, Germany

^2^ Jena School for Microbial Communication, Jena, 07743, Germany

^*^To whom correspondence should be addressed: michiel.kwantes@uni-jena.de and thomas.wichard@uni-jena.de (Fax: +493641948171)

Content:

Table S1: Oligomers used for adapter ligation and PCR

Table S2: Overview and taxonomic classification of the species surveyed in this study and corresponding data sources

Figure S1: Characterization of the pPIBT7 insertion site in locus *UM033_0004*.

Figure S2: Exon-intron structure of locus *UM003_0200* and corresponding protein domain architecture.

Table S1:

| **Table S1** Oligomers used for adapter ligation and PCR | method | genotyping | genotyping | genotyping | genotyping | adapter ligation | adapter ligation | flanking region PCR 1 | flanking region PCR 1 | flanking region nested PCR | flanking region nested PCR | flanking region PCR 1 - alternate primer | flanking region nested PCR - alternate primer | 5' RACE PCR 1 | 5' RACE nested PCR | 5' RACE PCR 1 | 5' RACE nested PCR | 3' RACE | 3' RACE | qPCR reference gene | qPCR reference gene | qPCR reference gene | qPCR reference gene | qPCR target gene | qPCR target gene and 3' insert verification with p34 | qPCR target gene | qPCR target gene | 5' insert verification in combination with p52 | 5' insert verification in combination with p34 | 3' insert verification in combination with p52 |
| --- | --- | --- | --- | --- | --- | --- | --- | --- | --- | --- | --- | --- | --- | --- | --- | --- | --- | --- | --- | --- | --- | --- | --- | --- | --- | --- | --- | --- | --- | --- |
|  | primer sequence (5'->3') | TTAGTCCTGCTCCTCGTCCACGAAGTG | GGCTTTCCAGGAGGGCAGTC | TACGGAGGCTGTTGCACACG | AGCAGCATGACAGACCATCTTGTG | CTAATACGACTCACTATAGGGCCAGCACTTGTCACTGAGGGCAGGT | GATCACCTGCCC | GAATCCTAATACGACTCACTATAGGGC | TGATGCGGTATTTTCTCCTTACGCATCTG | GCCAGCACTTGTCACTGAGG | TGTGCGCGGAACCCCTATTTG | TCACCAGCGTTTCTGGGTGAG | CAGGAAGGCAAAATGCCGCA | GGATGCTTGACACACTCCGGAAG | GTAGCCACTATCGAGCGCAGC | CCCACTGCTACTTTCATAGGAATGCTGGTTGCGAAC | GCTGCATTGCCTCCAGTTGTCCTTCGTG | GATTGCACGGAAATCGAGTTCTGT | GACGCTCAAGATATGTTTTAACCG | CCCTCGAAGTGGAGTCTTCTGAC | AAGTGTGCGGCCATCCTCTA | GGCAACTGCAGGAGCAATTCT | CCTCAGAAGCAACCTCGACCAT | GCTGCGCTCGATAGTGGCTAC | GGATGCTTGACACACTCCGGAAG | GGTAGCACAGGGGACATGCT | GATGGCAGCCGGAAGAAAGC | ACTCAGAGCGTGCTGCTTCGATC | CGGATTGGACCGCCTCTGTATC | CCTTGTTGCCGATTGGCGTAC |
|  | target identifier | pPIBT7 ble expression cassette | pPIBT7 ble expression cassette | *UM008_0183* (Ubiquitin) | *UM008_0183* (Ubiquitin) | adapter oligo | adapter oligo - 3' Aminolink C7 and 5' Phosphate modification | anneals to adapter | anneals to pPIBT7 vector sequence | anneals to adapter | anneals to pPIBT7 vector sequence | anneals to pPIBT7 vector sequence | anneals to pPIBT7 vector sequence | *UM033_0004* | *UM033_0004* | *UM005_0337* | *UM005_0337* | *UM033_0004* | *UM005_0337* | *UM008_0183* (Ubiquitin) | *UM008_0183* (Ubiquitin) | *UM010_0003* (PP2A 65 kDa regulatory subunit A) | *UM010_0003* (PP2A 65 kDa regulatory subunit A) | *UM033_0004* | *UM033_0004* | *UM005_0337* | *UM005_0337* | *UM033_0004* | *UM005_0337* | *UM005_0337* |
|  | name | p11 | p13 | p15 | p32 | p46 | p48 | p39 | p30 | p47 | p34 | p52 | p43 | p106 | p437 | p58 | p57 | p463 | p462 | p111 | p112 | p173 | p160 | p105 | p106 | p454 | p455 | p108 | p54 | p53 |

| **Table S2** Overview and taxonomic classification of the species surveyed in this study and corresponding data sources | taxonomic classification^c^ | order | Chloropicales | Picocystales | Mamiellales | Mamiellales | Mamiellales | Mamiellales | Mamiellales | Mamiellales | Chlorodendrales | Trebouxiales | Trebouxiales | Trebouxiophyceae ordo incertae sedis | Trebouxiales | Chlorellelales | Chlorellelales | Chlorellelales | Chlorellelales | Chlorellelales | Chlorellelales | Chlorellelales | Chlamydomonadales | Chlamydomonadales | Chlamydomonadales | Chlamydomonadales | Chlamydomonadales | Chlamydomonadales | Sphaeropleales | Sphaeropleales | Sphaeropleales | Sphaeropleales | Sphaeropleales | Sphaeropleales | Ulvales | Bryopsidales | Charales | Chlorokybales | Klebsormidales | Mesostigmatales | Mesostigmatales | Mesostigmatales |
| --- | --- | --- | --- | --- | --- | --- | --- | --- | --- | --- | --- | --- | --- | --- | --- | --- | --- | --- | --- | --- | --- | --- | --- | --- | --- | --- | --- | --- | --- | --- | --- | --- | --- | --- | --- | --- | --- | --- | --- | --- | --- | --- |
|  |  | class | Chloropicophyceae | Picocystophyceae | Mamiellophyceae | Mamiellophyceae | Mamiellophyceae | Mamiellophyceae | Mamiellophyceae | Mamiellophyceae | Chlorodendrophyceae | Trebouxiophyceae | Trebouxiophyceae | Trebouxiophyceae | Trebouxiophyceae | Trebouxiophyceae | Trebouxiophyceae | Trebouxiophyceae | Trebouxiophyceae | Trebouxiophyceae | Trebouxiophyceae | Trebouxiophyceae | Chlorophyceae | Chlorophyceae | Chlorophyceae | Chlorophyceae | Chlorophyceae | Chlorophyceae | Chlorophyceae | Chlorophyceae | Chlorophyceae | Chlorophyceae | Chlorophyceae | Chlorophyceae | Ulvophyceae | Ulvophyceae | Charophyceae | Chlorokybophyceae | Klebsormidiophyceae | Mesostigmatophyceae | Mesostigmatophyceae | Mesostigmatophyceae |
|  |  | phylum | Chlorophyta | Chlorophyta | Chlorophyta | Chlorophyta | Chlorophyta | Chlorophyta | Chlorophyta | Chlorophyta | Chlorophyta | Chlorophyta | Chlorophyta | Chlorophyta | Chlorophyta | Chlorophyta | Chlorophyta | Chlorophyta | Chlorophyta | Chlorophyta | Chlorophyta | Chlorophyta | Chlorophyta | Chlorophyta | Chlorophyta | Chlorophyta | Chlorophyta | Chlorophyta | Chlorophyta | Chlorophyta | Chlorophyta | Chlorophyta | Chlorophyta | Chlorophyta | Chlorophyta | Chlorophyta | Streptophyta | Streptophyta | Streptophyta | Streptophyta | Streptophyta | Streptophyta |
|  | data source^b^ | | phycocosm | phycocosm | phycocosm | phycocosm | phycocosm | phycocosm | phycocosm | phycocosm | phycocosm | phycocosm | phycocosm | phycocosm | phycocosm | phycocosm | phycocosm | phycocosm | phycocosm | phycocosm | phycocosm | phycocosm | phycocosm | phycocosm | phycocosm | phycocosm | phycocosm | phycocosm | phycocosm | phycocosm | phycocosm | phycocosm | phycocosm | phycocosm | phycocosm | phycocosm | phycocosm | phycocosm | phycocosm | phycocosm | phycocosm | phycocosm |
|  | Publication | | Lemieux C et al., 2019 | Junkins EN et al., 2019 | Moreau H et al., 2012 | Worden AZ et al., 2009 | Worden AZ et al., 2009 | Palenik B et al., 2007 | Blanc-Mathieu R et al., 2017 | Blanc-Mathieu R et al., 2014 | Steadman Tyler CR et al., 2019 | Armaleo D et al., 2019 | Browne DR et al., 2017 | Blanc G et al., 2012 | Greshake Tzovaras B et al., 2020 | Gao C et al., 2014 | Hamada M et al., 2018 | Arriola MB et al., 2018 | Blanc G et al., 2010 | Arriola MB et al., 2018 | Dahlin LR et al., 2019 | Gonzalez-Esquer CR et al., 2018 | Hirooka S et al., 2017 | Merchant SS et al., 2007 | Polle JEW et al., 2017 | Hanschen ER et al., 2016 | Featherston J et al., 2018 | Prochnik SE et al., 2010 | Roth MS et al., 2017 | Bogen C et al., 2013 | Suzuki S et al., 2018 | Carreres BM et al., 2017 | Starkenburg SR et al., 2017 | Calhoun S et al., 2021 | De Clerck O et al., 2018 | Arimoto A et al., 2019 | Nishiyama T et al., 2018 | Wang S et al., 2020 | Hori K et al., 2014 | Wang S et al., 2020 | Liang Z et al., 2020 | Cheng S et al., 2019 |
|  | Species name^a^ | | *Chloropicon primus* CCMP1205 | *Picocystis* sp. ML | *Bathycoccus prasinos* RCC1105 | *Micromonas commoda* NOUM17 (RCC 299) | *Micromonas pusilla* CCMP1545 | *Ostreococcus lucimarinus* | *Ostreococcus tauri* RCC1115 v1.0 | *Ostreococcus taur*i RCC4221 v3.0 | *Tetraselmis striata* | *Asterochloris glomerata* Cgr/DA1pho v2.0 | *Botryococcus braunii* Showa v2.1 | *Coccomyxa subellipsoidea* C-169 | *Trebouxia* sp. A1-2 | *Auxenochlorella protothecoide*s 0710 | *Chlorella* sp. A99 | *Chlorella sorokiniana* UTEX 1602 | *Chlorella variabili*s NC64A | *Micractinium conductrix* SAG 241.80 | *Picochlorum renovo* | *Picochlorum soloecismus* DOE101 | *Chlamydomonas eustigma* NIES-2499 | *Chlamydomonas reinhardtii* v5.6 | *Dunaliella salina* CCAP19/18 | *Gonium pectorale* NIES-2863 | *Tetrabaena socialis* NIES-571 | *Volvox carteri* v2.1 | *Chromochloris zofingiensis* SAG 211-14 | *Monoraphidium neglectum* SAG 48.87 | *Raphidocelis subcapitata* NIES-35 | *Scenedesmus obliquus* UTEX 393 | *Scenedesmus obliquus* UTEX B 3031 | *Scenedesmus* sp. NREL 46B-D3 v1.0 | *Ulva mutabilis* Føyn | *Caulerpa lentillifera* | *Chara braunii* S276 | *Chlorokybus atmophyticus* CCAC 0220 | *Klebsormidium nitens* NIES-2285 | *Mesostigma viride* CCAC 1140 | *Mesostigma viride* NIES-296 | *Mesotaenium endlicherianum* SAG 12.97 |

Table S2

|  | Brassicales | Marchantiaceae | Funariales | Sphagnaceae | Prasinodermatales | Glaucocystales | Gigartinales | Gracilariales | Cyanidiales | Cyanidiales | Cyanidiales | Cyanidiales | Cyanidiales | Cyanidiales | Cyanidiales | Cyanidiales | Cyanidiales | Cyanidiales | Cyanidiales | Cyanidiales | Bangiales | Bangiales | Gloeoemargaritales | ^a^If available species accession or genome version are given | ^b^The Phycocosm species and publication information was taken from https://phycocosm.jgi.doe.gov/archaeplastida/archaeplastida.info.html (accessed 26 March 2021) | ^c^Algae taxonomy according to Algaebase |
| --- | --- | --- | --- | --- | --- | --- | --- | --- | --- | --- | --- | --- | --- | --- | --- | --- | --- | --- | --- | --- | --- | --- | --- | --- | --- | --- |
|  | Magnoliopsida | Marchantiales | Bryopsida | Sphagnopsida | Prasinodermatophyceae | Glaucophyceae | Florideophyceae | Florideophyceae | Cyanidiophyceae | Cyanidiophyceae | Cyanidiophyceae | Cyanidiophyceae | Cyanidiophyceae | Cyanidiophyceae | Cyanidiophyceae | Cyanidiophyceae | Cyanidiophyceae | Cyanidiophyceae | Cyanidiophyceae | Cyanidiophyceae | Bangiophyceae | Bangiophyceae | Cyanophyceae |  |  |  |
|  | Streptophyta | Streptophyta | Streptophyta | Streptophyta | Prasinodermaphyta | Glaucophyta | Rhodophyta | Rhodophyta | Rhodophyta | Rhodophyta | Rhodophyta | Rhodophyta | Rhodophyta | Rhodophyta | Rhodophyta | Rhodophyta | Rhodophyta | Rhodophyta | Rhodophyta | Rhodophyta | Rhodophyta | Rhodophyta | Cyanobacteria |  |  |  |
|  | phytozome V12 | phytozome V12 | phytozome V12 | phytozome V12 | phycocosm | phycocosm | phycocosm | phycocosm | phycocosm | phycocosm | phycocosm | phycocosm | phycocosm | phycocosm | phycocosm | phycocosm | phycocosm | phycocosm | phycocosm | phycocosm | phycocosm | phycocosm | NCBI |  |  |  |
|  | Arabidopsis Genome Initiative, 2000 | Bowman JL et al., 2017 | Rensing SA et al., 2008 | Shaw AJ et al., 2016 | Li L et al., 2020 | Price DC et al., 2019 | Collén J et al., 2013 | Lee J et al., 2018 | Rossoni AW et al., 2019 | Nozaki H et al., 2007 | Rossoni AW et al., 2019 | Rossoni AW et al., 2019 | Schönknecht G et al., 2013 | Rossoni AW et al., 2019 | Rossoni AW et al., 2019 | Rossoni AW et al., 2019 | Rossoni AW et al., 2019 | Rossoni AW et al., 2019 | Rossoni AW et al., 2019 | Rossoni AW et al., 2019 | Brawley SH et al., 2017 | Nakamura Y et al., N/D | Ponce-Toledo RI et al., 2017 |  |  |  |
| **Table S2** (continued) | *Arabidopsis thaliana* Col-0 | *Marchantia polymorpha* v3.1 | *Physcomirella patens* v3.3 | *Sphagnum fallax* v0.5 | *Prasinoderma coloniale* CCMP1413 | *Cyanophora paradoxa* CCMP329 | *Chondrus crispus* Stackhouse | *Gracilariopsis chorda* isolate SKKU-2015 | *Cyanidioschyzon merolae* Soos | *Cyanidioschyzon merolae* strain 10D | *Galdieria phelgrea* Soos | *Galdieria sulphuraria* 002 | *Galdieria sulphuraria* 074W | *Galdieria sulphuraria* 5572 | *Galdieria sulphuraria* Azora | *Galdieria sulphuraria* MS1 | *Galdieria sulphuraria* MtSh | *Galdieria sulphuraria* RT22 | *Galdieria sulphuraria* SAG 21.92 | *Galdieria sulphuraria* YNP5578.1 | *Porphyra umbilicalis* isolate 4086291 | *Pyropia yezoensis* U-51 | *Gloeomargarita lithophora* Alchichica-D10 |  |  |  |

**Figure S1**

**
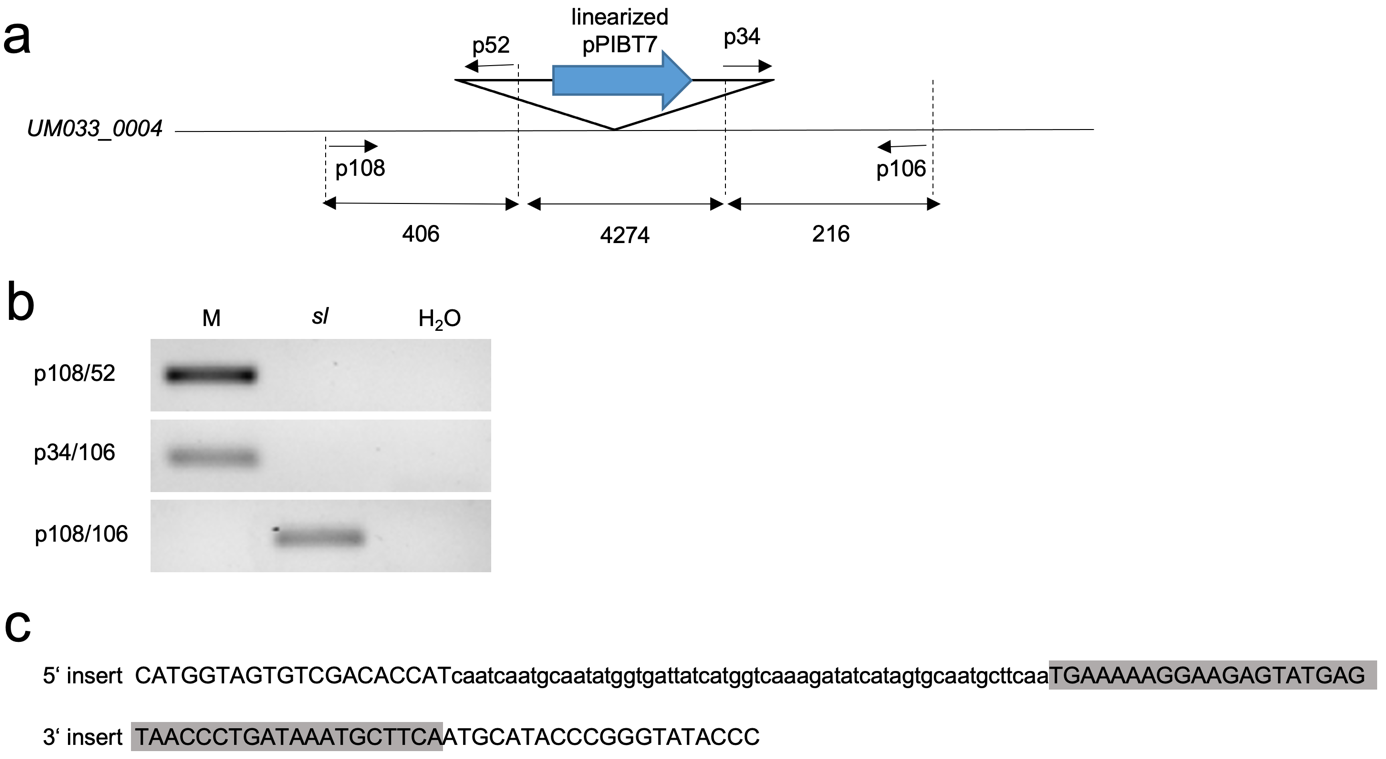
**

**Fig. S1** Characterization of the pPIBT7 insertion site in locus *UM033_0004*. **a** graphical representation of the insertion site. The triangle represents the inserted pPIBT7 sequence with the *ble* resistance cassette (block arrow) located in *UM003_0004* in the forward orientation. Arrows represent genotyping primers and double arrows represent the lengths (in basepair) of the depicted sequences. The sequence is not drawn to scale. **b** PCR analysis of the insertion site using the primers shown in (**a**). Sample M contained gDNA extracted from the *UM033_0004* insertional mutant and sample *sl* from the parent line *U. mutabilis* "*slender* ". **c** Sequence of the borders between the insert and the genomic DNA as determined by Sanger sequencing of the genotyping amplicons. Capital letters represent genomic sequence and shaded letters pPIBT7 sequence. On the 5' side of the pPIBT7 insert, 2 bp from the expected SspI linearized sequence are deleted, and there is a 57bp insertion of unknown origin (lowercase letters) between the vector and genomic DNA sequence. On the 3' side of the pPIBT7 insert, 5 bp from the expected SspI linearized pPIBT7 sequence are deleted.

**Figure S2**


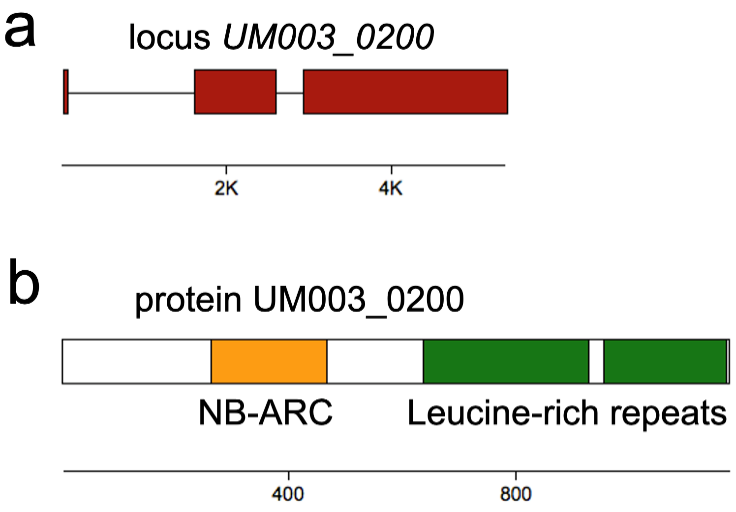


**Fig. S2** Exon-intron structure of locus *UM003_0200* and corresponding protein domain architecture. **a** Exon-intron structure of locus *UM003_0200*. Boxes represent exons and lines introns. The ruler shows tick marks every 2000 bases. **b** Protein domain architecture of UM003_0200. The ruler shows tick marks for every 400 amino acid residues.
